# Management of Osmoprotectant Uptake Hierarchy in *Bacillus subtilis* via a SigB-Dependent Antisense RNA

**DOI:** 10.3389/fmicb.2020.00622

**Published:** 2020-04-21

**Authors:** Hermann Rath, Alexander Reder, Tamara Hoffmann, Elke Hammer, Andreas Seubert, Erhard Bremer, Uwe Völker, Ulrike Mäder

**Affiliations:** ^1^Interfaculty Institute for Genetics and Functional Genomics, University Medicine Greifswald, Greifswald, Germany; ^2^Laboratory for Microbiology, Department of Biology, Philipps-University Marburg, Marburg, Germany; ^3^Faculty of Chemistry, Analytical Chemistry, Philipps-University Marburg, Marburg, Germany; ^4^Institute of Marine Biotechnology e.V. (IMaB), Greifswald, Germany

**Keywords:** antisense RNA, SigB, stress response, osmostress protectants, *Bacillus subtilis*

## Abstract

Under hyperosmotic conditions, bacteria accumulate compatible solutes through synthesis or import. *Bacillus subtilis* imports a large set of osmostress protectants via five osmotically controlled transport systems (OpuA to OpuE). Biosynthesis of the particularly effective osmoprotectant glycine betaine requires the exogenous supply of choline. While OpuB is rather specific for choline, OpuC imports a broad spectrum of compatible solutes, including choline and glycine betaine. One previously mapped antisense RNA of *B. subtilis*, S1290, exhibits strong and transient expression in response to a suddenly imposed salt stress. It covers the coding region of the *opuB* operon and is expressed from a strictly SigB-dependent promoter. By inactivation of this promoter and analysis of *opuB* and *opuC* transcript levels, we discovered a time-delayed osmotic induction of *opuB* that crucially depends on the S1290 antisense RNA and on the degree of the imposed osmotic stress. Time-delayed osmotic induction of *opuB* is apparently caused by transcriptional interference of RNA-polymerase complexes driving synthesis of the converging *opuB* and S1290 mRNAs. When our data are viewed in an ecophysiological framework, it appears that during the early adjustment phase of *B. subtilis* to acute osmotic stress, the cell prefers to initially rely on the transport activity of the promiscuous OpuC system and only subsequently fully induces *opuB*. Our data also reveal an integration of osmostress-specific adjustment systems with the SigB-controlled general stress response at a deeper level than previously appreciated.

## Introduction

Caused by periods of flooding and desiccation, the soil bacterium *Bacillus subtilis* is subjected to frequent changes in environmental osmolarity. An increase in external osmolarity triggers water efflux from the cell, causes dehydration of the cytoplasm, and a concomitant reduction in vital turgor pressure. Growth is thus impaired (Hoffmann and Bremer, 2017). To counteract these detrimental effects, cells initially take up potassium ions as an emergency stress reaction (Whatmore and Reed, 1990) and subsequently replace this ion with physiologically compliant organic osmolytes, the compatible solutes (Hoffmann and Bremer, 2017). *B. subtilis* can use at least 15 naturally occurring compatible solutes to provide osmotic stress resistance (Hoffmann and Bremer, 2017), of which proline is the only one it can synthesize *de novo* (Whatmore et al., 1990; Brill et al., 2011). In addition, *B. subtilis* can also synthesize glycine betaine, the probably most widely used compatible solute in nature (Yancey, 2005), provided that its biosynthetic precursor, choline, can be scavenged from external sources (Boch et al., 1994, 1996).

**FIGURE 1:**
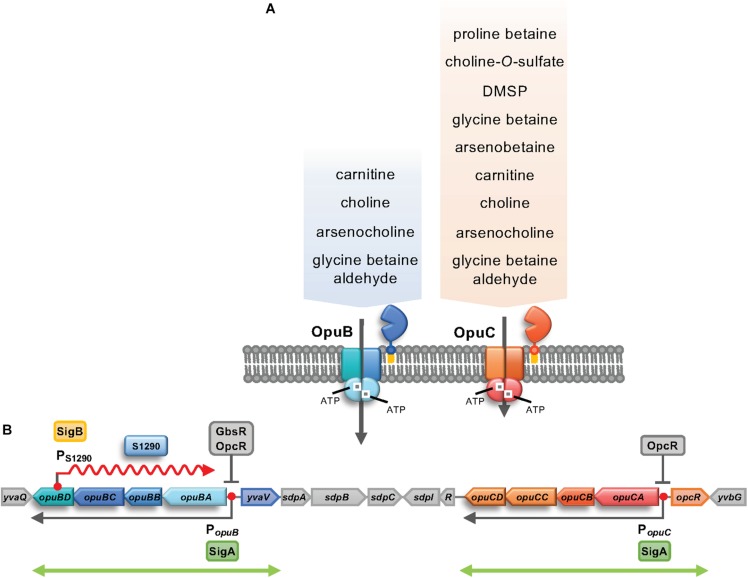
**(A)** Subunit composition and substrate profile of the OpuB and OpuC ABC transport systems. The OpuB and OpuC transporters consist of a homo-dimeric ATPase (OpuBA/OpuCA), hetero-dimeric *trans-*membrane components (OpuBB/OpuBD; OpuCB/OpuCD), and an extracellular substrate binding protein (OpuBC/OpuCC) that is tethered to the outer face of the cytoplasmic membrane via a lipid modification at its N-terminus (Kappes et al., 1999; Hoffmann and Bremer, 2017). The entire substrate spectrum of the OpuB system is shown, while only some of the 15 known substrates of the OpuC transporter are indicated (Hoffmann and Bremer, 2017; Teichmann et al., 2018). **(B)** Genetic organization of the *opuB* and *opuC* operons and their flanking regions. The positions of the SigA-dependent *opuB* and *opuC* promoters are indicated by red dots and the extent of the coding mRNA of these operons is depicted by arrows (Kappes et al., 1999). *opuB* expression is controlled by the repressor GbsR (Nau-Wagner et al., 2012) and by OpcR, while *opuC* expression is just responsive to OpcR (Lee et al., 2013). *opcR* and *yvaV* encode GbsR-type transcriptional regulators (Lee et al., 2013; Ronzheimer et al., 2018). The genes between *yvaV* and *opuCD* encode components of the *B. subtilis* cannibalism system and its genetic regulation (Gonzalez-Pastor et al., 2003; Ellermeier et al., 2006). The position of the SigB-dependent promoter in *opuBD* is given, as is the extent of the resulting S1290 asRNA annotated by Nicolas et al. (2012). The double-headed green arrows depict the duplicated region in the *B. subtilis* genome (Kunst et al., 1997; Kappes et al., 1999) that led to the evolution of the *B. subtilis* OpuB and OpuC transport systems (Teichmann et al., 2017).

*Bacillus subtilis* uses five osmotically regulated osmostress protectants uptake systems, the Opu transporters, to import the various compatible solutes. OpuA, OpuB and OpuC are high-affinity ABC transporters (Hoffmann and Bremer, 2017), OpuD belongs to the betaine-choline-carnitine-transporter (BCCT) family (Kappes et al., 1996; Ziegler et al., 2010), and the proline transporter OpuE is a member of the sodium-solute-symporter (SSS) family (von Blohn et al., 1997). Particularly effective osmostress protection is conferred by glycine betaine that can either be taken up from the environment via OpuA, OpuC and OpuD or synthesized by the GbsAB enzymes from the precursor choline imported via OpuB and OpuC (Boch et al., 1994; Kappes et al., 1996). Despite the fact that the OpuB and OpuC transporters are closely related by having evolved through a gene duplication event (Kunst et al., 1997; Kappes et al., 1999; Teichmann et al., 2017), the two ABC transporters possess strikingly different substrate specificities. OpuB exhibits a rather restricted substrate profile; it imports choline and arsenocholine with high affinity and carnitine with low affinity. In contrast, OpuC imports a broad range of osmoprotectants mostly with high affinity, including choline, arsenocholine, glycine betaine and arsenobetaine (Figure 1A; Hoffmann and Bremer, 2011, 2017; Hoffmann et al., 2018).

The choline/arsenocholine-sensing repressor GbsR controls the expression of the *gbsAB* operon encoding the enzymes that convert choline to glycine betaine and of the *opuB* operon, but not of the operon encoding the broad-spectrum OpuC transporter (Nau-Wagner et al., 2012; Hoffmann et al., 2018). Binding of intracellular choline/arsenocholine to GbsR releases this protein from its operator sites, and consequently results in enhanced *opuB* and *gbsAB* expression. The intracellular accumulation of glycine betaine/arsenobetaine counteracts the choline-mediated release of GbsR from the *opuB* and *gbsAB* operator sequences, thereby preventing excessive accumulation of glycine betaine by osmotically stressed *B. subtilis* cells (Nau-Wagner et al., 2012).

Expression of the three operons encoding the OpuA, OpuB and OpuC osmostress protectant ABC transporters and of the *opuD* and *opuE* genes is regulated at the transcriptional level by means of osmotically inducible SigA-type promoters (Kempf and Bremer, 1995; Kappes et al., 1996, 1999; Spiegelhalter and Bremer, 1998; Hoffmann et al., 2013). However, the *opuD* and *opuE* genes each possess a second promoter that is recognized by the general stress sigma factor SigB (Spiegelhalter and Bremer, 1998; Nicolas et al., 2012), thereby providing a genetic link between two adjustment systems to counteract osmotic stress.

In addition to stress-specific responses (e.g., the accumulation of compatible solutes), adaptation of *B. subtilis* to unfavorable conditions also involves the general stress response triggered by a variety of environmental and cellular cues. By coordinating the transcription of more than 200 genes (Nannapaneni et al., 2012; Nicolas et al., 2012), activation of the SigB general stress sigma factor provides *B. subtilis* with a nonspecific and preemptive multiple stress resistance (reviewed in Hecker et al., 2007). SigB is kept inactive by its anti-sigma factor RsbW and is released from the SigB/RsbW complex by the unphosphorylated form of the anti-anti sigma factor RsbV (Alper et al., 1996; Yang et al., 1996). RsbV is dephosphorylated by either of two phosphatases, RsbU and RsbP. Environmental stress signals are transduced by a large multiprotein complex, the stressosome, that controls the activity of RsbT (reviewed in Marles-Wright and Lewis, 2008), the positive regulator of the phosphatase RsbU (Völker et al., 1995; Yang et al., 1996). In contrast, energy stress signals are conveyed to SigB via RsbQ and the phosphatase RsbP (Vijay et al., 2000; Brody et al., 2001). SigB activation by environmental stresses, including ethanol, salt and heat, is transient and depends on the rate at which the stress increases over time (Völker et al., 1995; Young et al., 2013; Cabeen et al., 2017).

Activation of SigB also causes downregulation of many genes (Price et al., 2001). These indirect effects can be mediated by transcriptional regulators expressed from SigB-dependent promoters. Interestingly, a SigB-dependent antisense RNA (asRNA) was recently shown to be responsible for downregulation of *rpsD* encoding the ribosomal protein S4 upon exposure of *B. subtilis* to ethanol, resulting in lower levels of the small ribosomal subunit (Mars et al., 2015). For a second SigB-dependent asRNA covering the *cwlO* gene encoding a peptidoglycan hydrolase, no clear biological function could be detected (Noone et al., 2014). Altogether eight experimentally confirmed sense-antisense interactions, including four toxin-antitoxin systems, have been reported for *B. subtilis* (Silvaggi et al., 2005; Eiamphungporn and Helmann, 2009; Durand et al., 2012; Jahn et al., 2012; Luo and Helmann, 2012; Müller et al., 2016; Reif et al., 2018).

asRNAs are regulatory RNA molecules transcribed from the opposite strand of protein-coding genes. They can regulate their sense mRNA counterparts by various mechanisms, which rely on either base-pairing interactions or transcription interference (for reviews see Thomason and Storz, 2010; Georg and Hess, 2018). In a comprehensive tiling array based transcriptome study of *B. subtilis* exposed to a wide range of environmental conditions, asRNAs were detected for 13% of all protein-coding genes (Nicolas et al., 2012).

One of the asRNAs of *B. subtilis*, S1290, is transcribed from a promoter located on the opposite strand of the *opuBD* gene and covers in essence the complete *opuB* operon. The promoter classification analysis by Nicolas et al. (2012) predicted that the S1290 promoter is recognized by SigB (Figure 1B). An interesting observation of the study by Nicolas et al. (2012) was the differential induction of the *opuB* and *opuC* operons in response to both, suddenly imposed and sustained high salinity. Specifically, 10 min after an osmotic upshift elicited by addition of 0.4 M NaCl, only the *opuC* operon was induced. In contrast, during prolonged growth in the presence of 1.2 M NaCl, expression of *opuB*, but not of *opuC*, was increased compared to the control culture without additional salt. Notably, the S1290 RNA was strongly induced 10 min after exposure to salt stress.

The strong up-regulation of the S1290 RNA under osmotic up-shock conditions (Nicolas et al., 2012) is quite intriguing. However, a potential physiological role of this asRNA in modulating the function of the OpuB transporter during cellular adaptation of *B. subtilis* to osmotic stress is unexplored. To address this issue, we performed time-resolved analyses of *opuB*, *opuC*, and S1290 transcript levels in response to rapid osmotic upshifts in *B. subtilis* wild-type and S1290 promoter mutant strains. This set of experiments revealed a time-delayed osmotic induction of *opuB* that strictly depends on the S1290 asRNA. Our findings indicate that this is caused by transcriptional interference. Collectively, our data suggest that the changes in the transcriptional profile of *opuB* likely allow osmotically stressed *B. subtilis* cells to adjust their compatible solute pool to the prevailing environmental circumstances.

## Results

### Expression of the *opuB* and *opuC* Operons and of the S1290 asRNA Under Conditions of Suddenly Imposed and Sustained Increases in Salinity

The *opuB* (*opuBA-opuBB-opuBC-opuBD*) and *opuC* (*opuCA-opuCB-opuCC-opuCD*) operons of *B. subtilis* encode closely related ABC transport systems with amino acid identities of their components ranging from 69 to 80% (Kappes et al., 1999). Probes for Northern blot analysis were designed against the 5′-part of the genes encoding the extracellular substrate-binding proteins of the ABC transporters [(*opuBC*; nucleotides 200–470) and (*opuCC*; nucleotides 17–519)], as these are the least conserved components of the OpuB and OpuC systems (Figure 1). In order to exclude any undesired cross-hybridization, the probes were tested against RNA samples of *B. subtilis* mutant strains in which one of the two operons is completely deleted. No hybridization signals were obtained in the respective deletion mutants (data not shown), demonstrating that these Northern blot probes can be used to specifically trace changes in the amounts of *opuB* and *opuC* mRNA, respectively.

**FIGURE 2:**
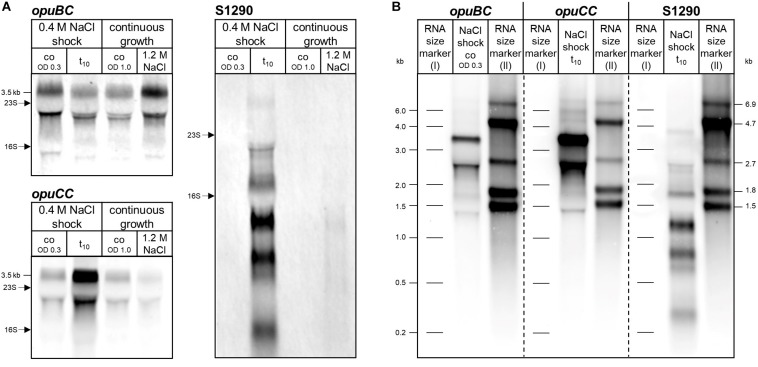
**(A)** Northern blot analysis of *opuBC*, *opuCC* and S1290 transcript levels in the *B. subtilis* wild-type strain BSB1 before (co) and 10 min (t_10_) after salt shock and during growth at high salinity (1.2 M NaCl) in comparison to SMM without additional salt (SMM). For each sample 5 μg of total RNA per lane were loaded. The size of the respective full-length transcripts and the positions of the 16S and 23S rRNA are indicated. **(B)** Determination of *opuBC*, *opuCC* and S1290 transcript sizes by Northern blot analysis using two RNA size markers. Fragment sizes of the markers are indicated next to the image. The following samples with high levels of the respective transcripts were used: for *opuBC*, before (co), for *opuCC* and S1290, 10 min (t_10_) after salt shock.

*opuB* and *opuC* expression under conditions of acute and sustained salt stress has previously been assessed in a comprehensive tiling array study (Nicolas et al., 2012). To critically re-evaluate these data, the *B. subtilis* wild-type strain BSB1 (168 Trp^+^) was grown in Spizizen’s minimal medium (SMM) until an optical density at 600 nm (OD_600_) of 0.3 was reached and then cells were stressed with 0.4 M NaCl for 10 min or grown in SMM in the absence or presence of NaCl (1.2 M) until reaching an OD_600_ of 1. Northern blot analysis with the *opuBC*- and *opuCC*-specific probes (Figure 2) detected the expected full-length transcripts (approximately 3.5 Kb). This experiment revealed that 10 min after an osmotic upshift elicited by addition of 0.4 M NaCl, transcription of the *opuC* operon was induced, whereas the *opuB* expression level was slightly lower compared to the sample taken before exposure to salt stress. Only prolonged growth in high-salinity medium led to increased expression of *opuB*. Under these conditions, cells exhibited basal levels of *opuC* expression. These results are in agreement with the data reported by Nicolas et al., 2012 and confirmed the different patterns of transcriptional induction of the *opuB* and *opuC* operons in response to either suddenly imposed or sustained high salinity.

The asRNA S1290 (Figure 1B) was identified by Nicolas et al. (2012) and its transcriptional profile suggested that its synthesis was presumably dependent on the alternative sigma factor SigB. Inspection of the expression pattern of S1290 asRNA in the dataset provided by Nicolas et al. (2012) revealed a strong induction under stress conditions typical for SigB regulated genes (Nannapaneni et al., 2012), including the sudden exposure to salt, ethanol or heat (see *B. subtilis* Expression Data Browser^1^). Following the canonical expression profile of SigB-regulated genes, the S1290 RNA was not detected under control conditions and during prolonged growth in high-salinity medium, but was strongly induced 10 min after imposition of salt stress (Figure 2), one of the strongest inducers of the entire SigB regulon (Petersohn et al., 2001). The 5′-end of S1290 resides in *opuBD*, the fourth gene of the *opuB* operon (Figure 1B and *B. subtilis* Expression Data Browser^2^). Using a probe complementary to nucleotides 51–351 of S1290, several transcripts were detected, with sizes of 0.3, 0.6, 0.75, 1.15, 1.7, and 3.8 Kb, of which the most prominent one (1.15 Kb) would end near the 5′-extremity of *opuBC*. The largest transcript most probably extends to the termination site of the downstream *yvaV* gene. Hence, the S1290 asRNA practically covers the entire coding region of the *opuB* operon and should interfere with *opuB* transcription or translation of the *opuB* mRNA.

**FIGURE 3:**
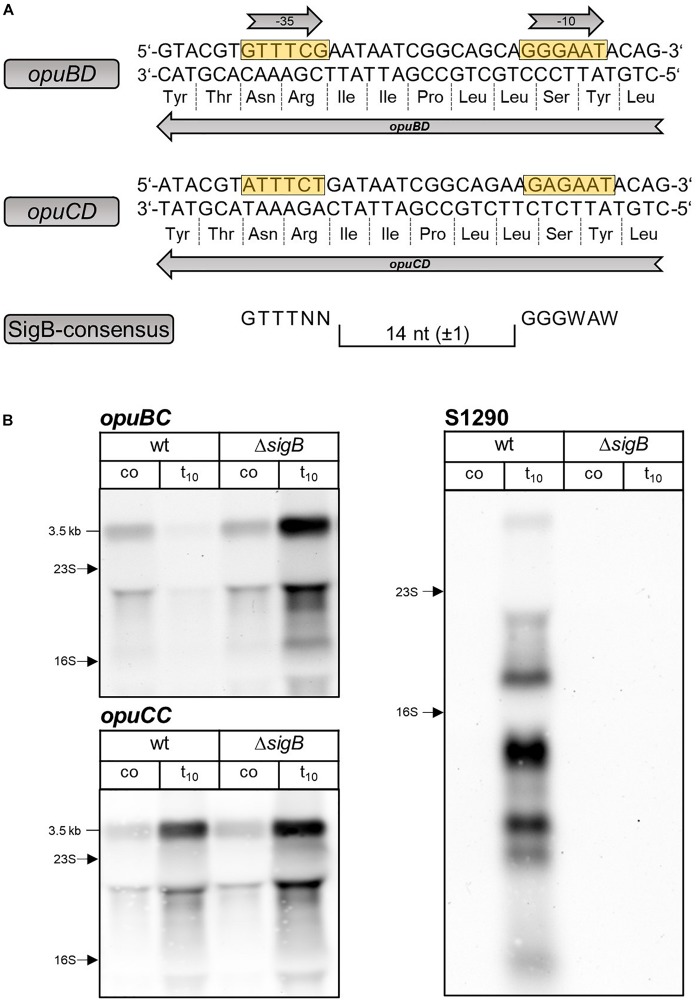
**(A)** Sequence of the *opuBD* region containing the S1290 promoter and the corresponding region of *opuCD*. The –35 and –10 sequences of the SigB-dependent promoter are boxed in yellow and the amino acid sequences of OpuBD and OpuCD are shown. The consensus sequence of SigB-type promoters is depicted below. **(B)** Analysis of *opuBC*, *opuCC* and S1290 transcript levels in wild-type (wt) and *sigB* mutant (Δ*sigB*) cells before (co) and 10 min (t_10_) after salt shock with 0.4 M NaCl. For each sample 5 μg of total RNA per lane were loaded. The size of the respective full-length transcripts and the positions of the 16S and 23S rRNA are indicated.

Inspection of the *opuBD* region immediately preceding the 5′-end of the S1290 RNA and the corresponding region of *opuCD* revealed the presence of a conserved SigB-type promoter (Petersohn et al., 2001) only in the case of the *opuBD* (Figure 3A). In contrast, the corresponding region of *opuCD* differs in two conserved positions of the promoter elements (A instead of G at position 1 of the −35 region and A instead of G at position 2 of the −10 region). To prove that SigB is responsible for stress induction of the S1290 promoter, S1290 levels were compared in the *B. subtilis* wild-type BSB1 and an isogenic *sigB* deletion mutant. In the strain lacking SigB, no S1290 RNA was observed after imposition of salt stress (Figure 3B). Strikingly, under these conditions *opuB* expression was induced already after 10 min of salt stress to the same level as that of *opuC*. This observation provided the first indication that osmotic induction of *opuB* expression might be affected by the SigB-controlled S1290 asRNA.

### Time-Resolved Expression of the *opuB* Operon and Its asRNA S1290 After Imposition of Salt Stress

**FIGURE 4:**
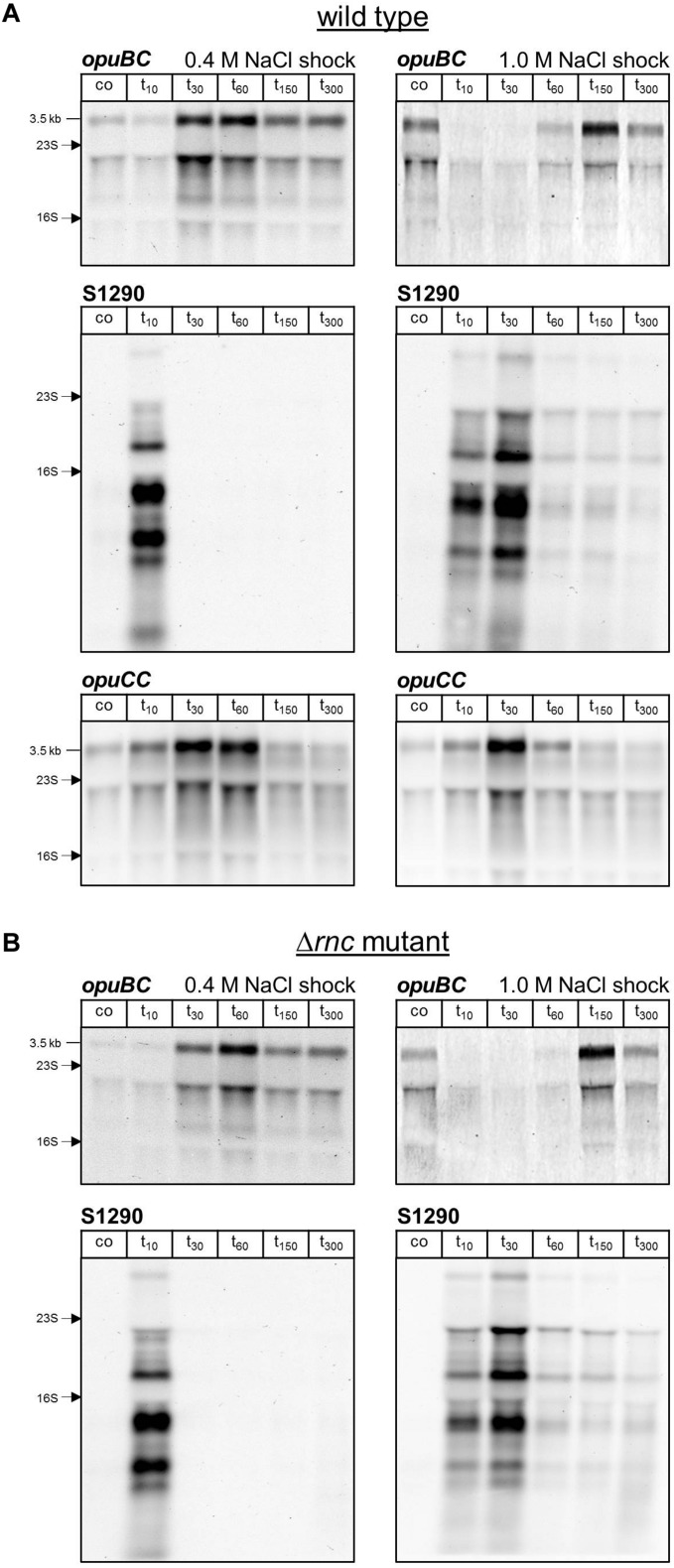
**(A)** Northern blot analysis of changes in *opuBC*, S1290 and *opuCC* transcript levels following salt shock with 0.4 M or 1.0 M NaCl in *B. subtilis* BSB1 wild-type. **(B)** Analysis of changes in *opuBC* and S1290 transcript levels following salt shock with 0.4 M or 1.0 M NaCl in *B. subtilis* BHR006 (Δ*rnc*). Cells were harvested before (co) and 10, 30, 60, 150, and 300 min after addition of salt. For each sample 5 μg of total RNA per lane were loaded. The size of the respective full-length transcripts and the positions of the 16S and 23S rRNA are indicated.

In order to analyze the relationship between expression of the *opuB* operon and the S1290 asRNA, we grew the *B. subtilis* wild-type strain BSB1 (168 Trp^+^) in SMM and challenged exponentially growing cells with either 0.4 M or 1 M NaCl. Samples for RNA preparation were withdrawn before and 10, 30, 60, 150, and 300 min after addition of NaCl. Because antisense-sense interactions can affect not only mRNA amounts, but also transcript patterns through mRNA processing or partial degradation (Georg and Hess, 2018), expression of S1290 and the *opu* genes was monitored by Northern blot analysis, allowing for quantitative measurements of relative RNA abundance and detection of individual RNA species with different sizes. This analysis revealed a delayed induction of the *opuB* operon after imposition of salt stress (Figure 4A). At the first time point analyzed (10 min), *opuB* transcript levels were more than 1.5-fold lower compared to pre-stress levels (Table 1). The earliest time points at which induced transcript levels could be observed were 30 min in the case of 0.4 M NaCl and 150 min in the case of 1 M NaCl. The S1290 asRNA showed an expression pattern inversely related to that of *opuB*. S1290 levels peaked at 10 or 30 min, respectively, after the osmotic upshift, which can be explained by the transient activation of SigB. This difference in the timing of S1290 expression, which depends on the severity of the osmotic stress, is in agreement with previous results on the speed and magnitude of SigB activity (Cabeen et al., 2017). In contrast to *opuB*, *opuC* expression responded to an osmotic upshift within 10 min and reached a maximum level at 30 min (Figure 4A). At subsequent time points, *opuC* mRNA gradually declined and returned to pre-stress levels in cells adapted to growth in high-salinity medium. The decline was more pronounced at the higher salt concentration of 1 M NaCl. The results obtained in this experiment suggest that expression of the S1290 asRNA retards the osmotic induction of the *opuB* operon.

**TABLE 1:** Relative *opuB* transcript levels at different time points after addition of 0.4 M NaCl.

*Strain*\*Time point*	*t*_10_	*t*_30_	*t*_60_	*t*_150_	*t*_300_
BSB1 wild-type	0.59 (±0.18)	3.49 (±0.24)	3.40 (±1.25)	1.91 (±0.27)	2.03 (±0.62)
P_*S*1290_ mutant	2.74 (±1.04)	3.68 (±1.58)	4.00 (±0.49)	1.34 (±0.60)	1.53 (±1.02)
Δ*gbsR* mutant	0.58 (±0.09)	2.94 (±0.69)	2.48 (±0.92)	1.29 (±0.08)	1.09 (±0.04)

*Transcript levels detected before the addition of salt were set to 1.0. Standard deviation of the Northern blot signals from two independent experiments is shown in brackets.*

### Inactivation of the S1290 SigB-Promoter Prevents Time-Delayed Induction of *opuB* After Osmotic Upshift

**FIGURE 5:**
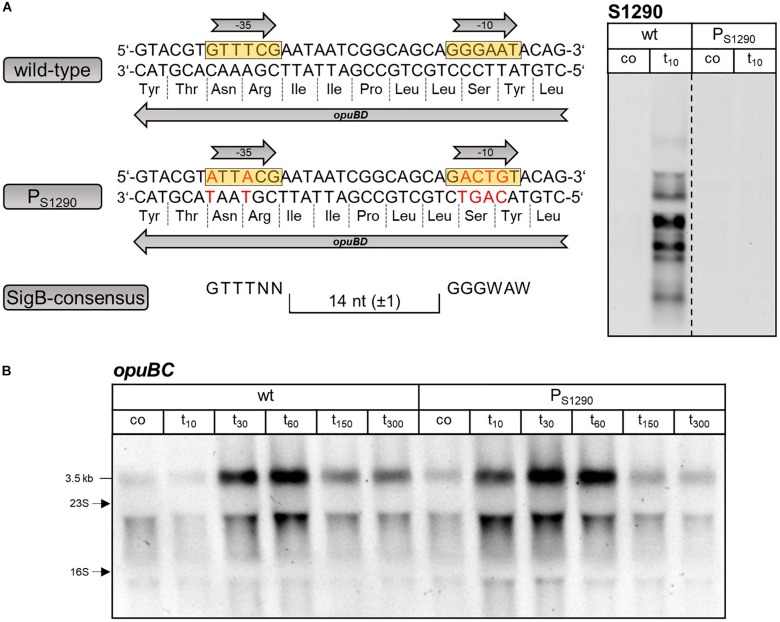
**(A)** Sequence of the S1290 promoter region in the *B. subtilis* wild-type and P_*S*1290_ mutant. The –35 and –10 sequences of the SigB-dependent promoter are boxed in yellow and the amino acid sequence of OpuBD is shown. The consensus sequence of SigB-type promoters is depicted below. The base substitutions introduced without changing the amino acid sequence of OpuBD in the P_*S*1290_ mutant are indicated by red letters. Northern blot analysis using the S1290 specific probe was performed before (co) and 10 min (t_10_) after salt shock with 0.4 M NaCl to verify the absence of S1290 induction in the P_*S*1290_ mutant. **(B)** Northern blot analysis of *opuBC* transcript levels in *B. subtilis* BSB1 (wt) and BHR010 (P_*S*1290_) before (co) and 10, 30, 60, 150, and 300 min after salt shock imposed by addition of 0.4 M NaCl. For each sample 5 μg of total RNA per lane were loaded. The size of the *opuB* full-length transcript and the positions of the 16S and 23S rRNA are indicated.

In order to verify that expression of the S1290 asRNA is indeed causative for the time-delayed induction of the *opuB* operon, we sought to construct a mutant in which only S1290 is no longer expressed. Because the S1290 promoter is located within the coding region of *opuBD* (Figure 1B), it was not possible to simply delete the promoter region. Therefore, the promoter was inactivated by altering essential positions of the −10 and −35 regions of the presumed SigB-dependent promoter without changing the amino acid sequence of the OpuBD protein (Figure 5A). In the resulting P_*S*1290_ promoter mutant, transcription of S1290 in response to salt stress was abolished. Importantly, Northern Blot analysis of a time-series experiment (samples taken before and 10, 30, 60, 150, and 300 min after addition of 0.4 M NaCl) showed that delayed upregulation of the *opuB* operon was no longer observed in the absence of S1290 (Figure 5B). In this strain, imposition of salt stress led to a more than two-fold increase in *opuB* mRNA levels within 10 min, which were further increased at the 30 min time point (Table 1). Comparable levels of *opuB* mRNA were detected 60 min after stress induction in wild-type and P_*S*1290_ mutant cells.

### Antisense Regulation of *opuB* Expression Depends on the Genomic Localization of S1290

**FIGURE 6:**
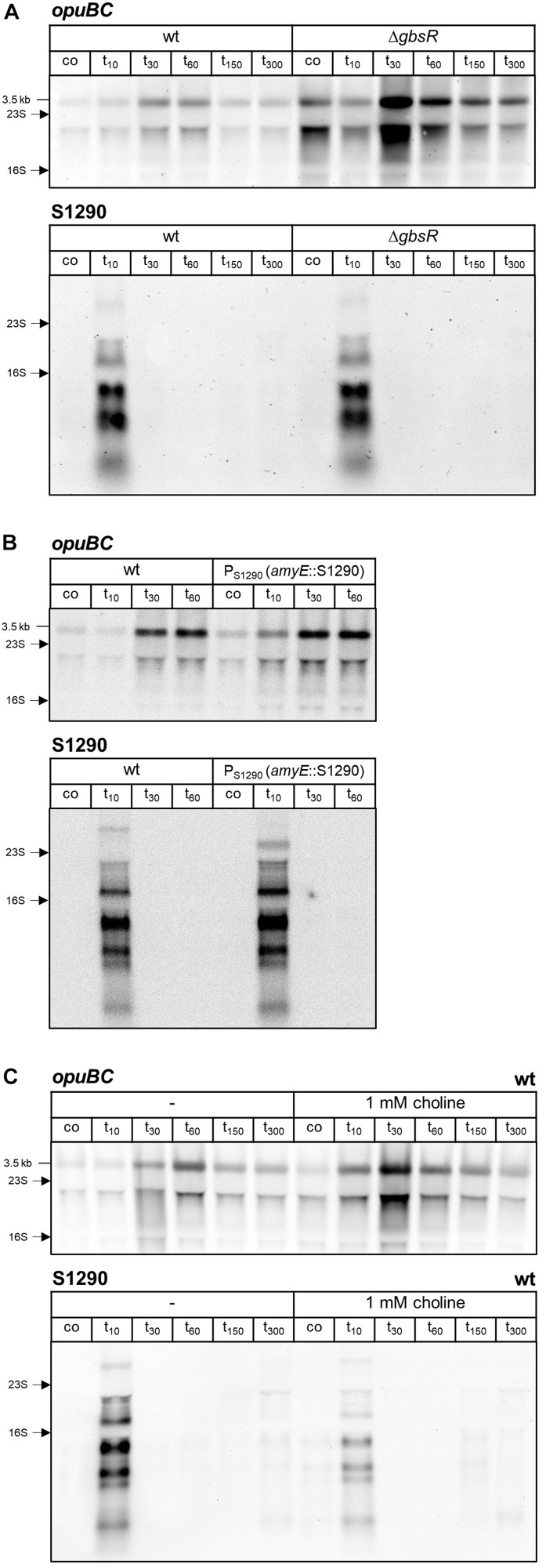
Northern blot analysis of *opuBC* and S1290 transcript levels in **(A)** *B. subtilis* BSB1 (wt) and TMB405 (Δ*gbsR*), **(B)** *B. subtilis* BSB1 (wt) and BHR018 (P_*S*1290_ *amyE*::S1290), and **(C)** *B. subtilis* BSB1 wild-type in the presence or absence of 1 mM choline. Cells were harvested before (co) and 10, 30, 60, 150, and 300 min after salt shock imposed by addition of 0.4 M NaCl. For each sample 5 μg of total RNA per lane were loaded. The size of the *opuB* full-length transcript and the positions of the 16S and 23S rRNA are indicated.

AsRNAs can regulate their sense mRNAs by various mechanisms, which rely on either base-pairing interactions or transcription interference (for reviews see Thomason and Storz, 2010; Georg and Hess, 2018). Formation of double-stranded RNA can trigger RNA degradation by providing a substrate for the RNase III endoribonuclease. The double-strand specific RNase III (encoded by *rnc*) was shown to play a role in the regulation of gene expression by asRNAs in Gram-positive bacteria (reviewed in Durand and Condon, 2018). Therefore, we investigated the impact of RNase III inactivation on *opuB* sense and antisense transcript levels. Because *rnc* can only be deleted in the absence of two prophage-encoded toxin genes (Durand et al., 2012), we constructed a Δ*txpA* Δ*yonT* Δ*rnc* mutant. Using this strain, we performed the same time-series analyses of *opuB* and S1290 transcript levels after adding 0.4 M or 1 M NaCl as described above for the wild-type strain (Figure 4A). The amounts of *opuB* mRNA and S1290 asRNA as well as the relative amounts of the individual S1290 species were not significantly different between the Δ*txpA* Δ*yonT* Δ*rnc* deletion mutant (Figure 4B) and the wild-type strain, ruling out that the asRNA exerts its effect on *opuB* expression via co-degradation of the RNA duplex by RNase III. This result suggested that antisense regulation of *opuB* occurs at the level of transcription, in that stress-induced transcription from the SigB-dependent S1290 promoter could suppress transcription of *opuB* by interference between convergent RNA polymerases. In support of this hypothesis, delayed induction of *opuB* expression in response to salt stress was also observed in a mutant lacking the transcriptional repressor (GbsR) of the *opuB* operon (Nau-Wagner et al., 2012), a strain, which accordingly exhibits higher *opuB* transcript levels even under non-inducing conditions (Figure 6A and Table 1).

Next, we tested whether antisense regulation of *opuB* is abolished when S1290 is expressed from a different genomic locus. To this end, a sequence containing the S1290 promoter and a 3 Kb region of the asRNA was inserted into the *amyE* gene of the P_*S*1290_ mutant. When S1290 is expressed in *trans*, the same antisense transcripts in similar amounts are detected as in wild-type cells (Figure 6B), of which only the largest transcript (3.3 Kb) is slightly shorter because the inserted sequence does not contain the *opuBA* promoter region and the *yvaV* gene. In the *trans* constellation, the increase in *opuB* mRNA abundance after imposition of salt stress was no longer delayed (Figure 6B) as it was the case in the absence of S1290 expression (Figure 5B). Hence, the S1290 asRNA can be assumed to control gene expression by interfering with transcription of the *opuB* operon rather than a mechanism that depends on asRNA-mRNA base pairing.

### Intracellular Glycine Betaine Accumulation Reduces S1290 Induction by Mitigating SigB Activation

Osmotic induction of *opuB* transcription and the choline-responsive release of the GbsR repressor from its operator are genetically separable events (Nau-Wagner et al., 2012). As expected, the Δ*gbsR* mutant exhibited both stronger *opuB* basal level expression as well as induction after imposition of salt stress as compared to the wild-type, with the highest level reached 30 min after the imposition of the salt shock (Figure 6A). However, salt induction was still delayed in the *gbsR* mutant and did not occur 10 min after addition of 0.4 M NaCl. Import of choline, the inducer for the GbsR repressor, also relieves GbsR-mediated repression of the *opuB* and *gbsAB* operons, thus enabling enhanced choline uptake and synthesis of glycine betaine (Nau-Wagner et al., 2012). Therefore, we expected a similar pattern of *opuB* induction elicited by salt stress when the wild-type was grown in SMM supplemented with choline as observed in the strain lacking the GbsR repressor. Indeed, 30 min after addition of 0.4 M NaCl, enhanced *opuB* expression level was detected when 1 mM choline was present in the medium (Figure 6C), data comparable to those obtained with the *gbsR* mutant. However, in contrast to the *gbsR* mutant, wild-type cells grown in the presence of choline showed an immediate increase in *opuB* transcript level already 10 min after salt shock. Strikingly, salt induction of the S1290 asRNA was strongly reduced under these conditions.

**FIGURE 7:**
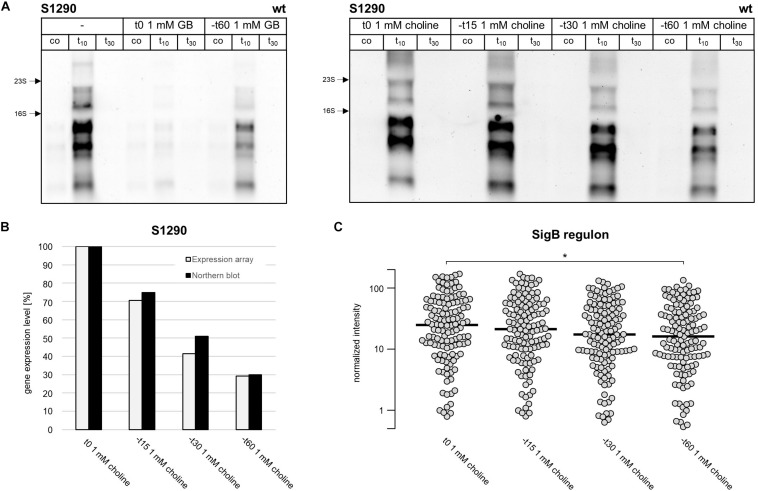
Intracellular glycine betaine accumulation reduces S1290 induction by alleviating activation of the SigB regulon **(A)** Northern blot analysis of S1290 transcript levels in the *B. subtilis* wild-type strain BSB1. Cells were harvested before (co) and 10 and 30 min after salt addition. Left panel: *B. subtilis* was grown without glycine betaine (–) or with glycine betaine added immediately or 60 min, respectively, before addition of 0.4 M NaCl. Right panel: Choline was added immediately or 15, 30, and 60 min, respectively, before addition of 0.4 M NaCl. **(B)** S1290 transcript levels measured by microarray analysis were compared to northern blot signal intensities. Choline was added immediately or 15, 30, and 60 min, respectively, before addition of 0.4 M NaCl. RNA for transcriptome analysis was isolated from cells harvested 10 min after salt addition. The data were normalized to the sample where choline was added immediately before salt addition. **(C)** Induction of the SigB regulon. Each dot represents a SigB-dependent gene and for each condition the median expression level over all genes is indicated as a black bar. Only genes classified as SigB-regulated by two independent studies (Nannapaneni et al., 2012; Nicolas et al., 2012) were considered. Difference in the induction level of the SigB regulon between individual time points was tested for statistical significance by use of a Student’s *T*-test (*p-value ≤ 0.05).

These observations led us to speculate that the intracellular accumulation of glycine betaine synthesized from choline reduces S1290 levels. In the presence of extracellular glycine betaine or choline, the intracellular glycine betaine pool of *B. subtilis* cells increases in tune with the increases in the salt concentration of the growth medium (Hoffmann et al., 2013). As noted before, glycine betaine accumulates to notable levels even in cells grown in SMM without additional NaCl, a process that is largely dependent on the substantial transport activity of the OpuA system even in the absence of notable osmotic stress in a standard minimal growth medium (Hoffmann et al., 2013). Our results (Figure 6C) thus indicate that these levels of glycine betaine in non salt-stressed cells can already affect induction of S1290 expression. Strikingly, addition of glycine betaine to salt-stressed cells completely prevented S1290 induction when added at the time of stress imposition (Figure 7A); this phenomenon is likely due to the fast accumulation of glycine betaine from exogenous sources in salt-stressed cells (Kappes et al., 1996; Hoffmann and Bremer, 2017). If glycine betaine was added 60 min before the osmotic upshift, S1290 RNA was clearly detectable, but – as observed when *B. subtilis* was grown in the presence of the glycine betaine precursor choline (Figure 6C) – the S1290 level was significantly reduced compared to conditions without added glycine betaine. If indeed the osmoprotectant glycine betaine is responsible for reduced S1290 levels, addition of choline to the culture at different times prior to the salt shock should have different effects on the appearance of the S1290 RNA, because choline needs to be first converted to glycine betaine to confer cellular osmostress protection (Boch et al., 1996). In support of this hypothesis, SigB-dependent S1290 levels were not reduced if choline was added to the cultures at the time of salt stress imposition, but gradually declined with increasing time that had passed (15, 30, 60 min) between the addition of choline prior to the salt shock (Figure 7A). Thus, intracellular accumulation of glycine betaine seems to influence signal perception by SigB, as assessed by S1290 levels under conditions of salt stress.

### Influence of Glycine Betaine on the Transcriptional Profile of the SigB Regulon

In order to further probe whether salt-stress dependent activation of SigB is mitigated when cells are pre-loaded with glycine betaine, we analyzed at a genome-wide level salt induction of the SigB regulon in the presence of choline using microarrays. The glycine betaine precursor choline was added to exponentially growing cultures either immediately, or 15, 30, and 60 min prior to the osmotic upshift imposed to all cultures at OD_600_ of 0.3. RNA was then isolated for transcriptome analysis from cells that were stressed with 0.4 M NaCl for 10 min. After sample processing and quantification of the hybridization signals, we first investigated S1290 RNA levels, thereby confirming that induction of S1290 decreased with increasing duration of presence of choline in the growth medium prior to the salt shock (Figure 7B). We then analyzed the expression levels of all known SigB-regulated genes (Nannapaneni et al., 2012; Nicolas et al., 2012; Supplementary Table S1). As depicted in Figure 7C, the SigB regulon showed the same pattern of gradual reduction in transcriptional induction with increasing duration of the presence of choline as observed for the S1290 asRNA. The median expression value of SigB-regulated genes was 1.7-fold higher in cells that received choline immediately before the salt stress compared to cells where choline was added 60 min prior to the salt stress.

### S1290-Mediated Delay of *opuB* Expression Diminishes Release of GbsR-Mediated Repression

**FIGURE 8:**
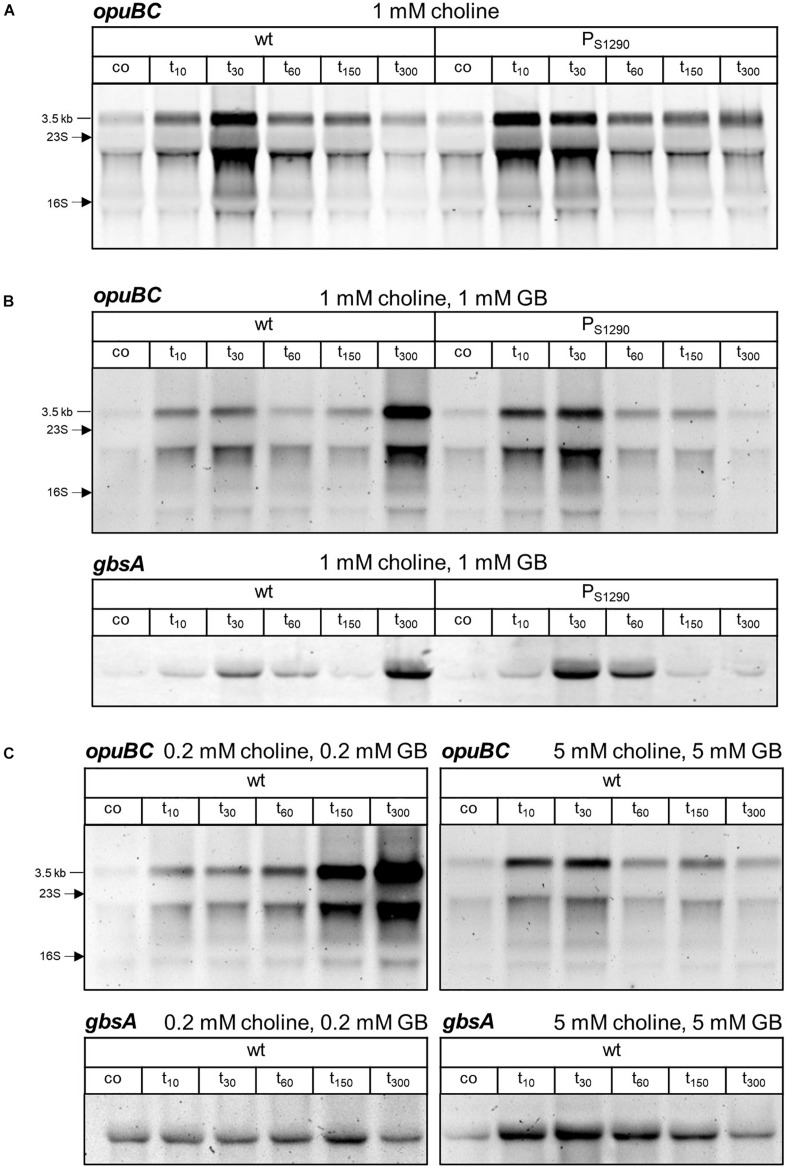
Northern blot analysis of **(A)** *opuBC* transcript levels in *B. subtilis* BSB1 (wt) compared to BHR010 (P_*S*1290_ mutant) in the presence of 1 mM choline and of **(B)** *opuBC* and *gbsAB* transcript levels in the presence of 1 mM choline/1 mM glycine betaine. **(C)** *OpuBC* transcript levels in *B. subtilis* BSB1 wild-type in the presence of either 0.2 mM choline/0.2 mM glycine betaine or 5 mM choline/5 mM glycine betaine. Cells were harvested before (co) and 10, 30, 60, 150 and 300 min after salt shock imposed by addition of 0.4 M NaCl. For each sample 5 μg of total RNA per lane were loaded. The size of the respective full-length transcripts and the positions of the 16S and 23S rRNA are indicated.

Next, we assessed if even reduced S1290 levels observed when *B. subtilis* is grown in the presence of choline can affect *opuB* expression after salt shock. To this end, we compared *opuB* induction in wild-type and P_*S*1290_ mutant cells cultivated in SMM containing 1 mM choline. Indeed, the moderate increase in *opuB* expression observed in the wild-type 10 min after addition of 0.4 M NaCl (Figures 6C, 8A) was significantly enhanced in the mutant lacking the S1290 asRNA, where the maximum level of *opuB* induction was already reached at this early time point (Figure 8A). Hence, expression of *opuB* after salt shock peaked earlier in the S1290 promoter mutant as compared to the wild-type, whereas the magnitude of induction reached under these conditions of choline-mediated release of GbsR remained unaffected.

In response to a sudden osmotic upshift, *B. subtilis* induces the expression of the *opuA*, *opuC* and *opuD* glycine betaine transporters genes (Nicolas et al., 2012; Hoffmann et al., 2013), thus accomplishing immediate uptake of glycine betaine. Intracellular glycine betaine counteracts the choline-mediated de-repression of the *opuB* and *gbsAB* operons through its influence on GbsR (Nau-Wagner et al., 2012). We hypothesized that in the presence of glycine betaine and choline in the medium, delayed *opuB* expression could result in an intracellular glycine betaine to choline ratio that prevents strong induction of *opuB* and *gbsAB* after salt shock. In order to test this hypothesis, we grew the *B. subtilis* wild-type and the P_*S*1290_ mutant strains in SMM containing 1 mM glycine betaine/1 mM choline and exposed exponentially growing cells to salt shock with 0.4 M NaCl. The lack of the S1290 asRNA in the P_*S*1290_ strain indeed led to stronger induction of the *opuB* and *gbsAB* operons (Figure 8B), which can be explained by increased choline import resulting in the removal of GbsR-mediated repression (Nau-Wagner et al., 2012). Thus, the asRNA-mediated delay in choline uptake prevents strong expression of *opuB* and *gbsAB* in *B. subtilis* wild-type cells and thereby reduces synthesis of proteins dedicated to choline uptake and conversion.

Another striking difference between the wild-type and the P_*S*1290_ mutant subjected to salt stress in the presence of 1 mM glycine betaine/1 mM choline was the de-repression of both *opuB* and *gbsAB* 300 min after the osmotic upshift in wild-type, but not in mutant cells (Figure 8B). Probably, the pool of glycine betaine provided in the medium had been exhausted by the wild-type cells before that time point, necessitating the uptake and conversion of choline to maintain the intracellular glycine betaine pool required for growth at 0.4 M NaCl. In the case of the P_*S*1290_ mutant, synthesis of glycine betaine from imported choline would reduce the consumption of externally provided glycine betaine, and indeed no de-repression of the GbsR-dependent genes occurred at 300 min, because sufficient amounts of glycine betaine seemed to be still available making choline uptake unnecessary. If this assumption is true, earlier de-repression of *opuB* and *gbsAB* should be observed if lower amounts of glycine betaine are provided in the medium und de-repression should be prevented by supply of excess glycine betaine, respectively. Indeed, when salt stress was applied to wild-type cells growing in SMM supplemented with a mixture of 0.2 mM glycine betaine/0.2 mM choline, increased *opuBC* and *gbsA* mRNA levels were already observed after 150 min (Figure 8C). Consistently, in SMM containing 5 mM glycine betaine/5 mM choline, recurrence of *opuB* and *gbsAB* induction was not observed within 300 min after the osmotic upshift.

## Discussion

In this study, we ascribe a physiological function to the long non-coding antisense RNA S1290 in the framework of the osmostress response of *B. subtilis*. asS1290 exerts its regulatory effect through its influence on the transcriptional profile of the *opuB* operon, an osmotically inducible gene cluster encoding the OpuB ABC transporter for the biosynthetic precursors for the osmostress protectants glycine betaine and arsenobetaine (Figure 1A). Physiologically, OpuB is an integral part of the central osmostress response network of *B. subtilis* relying on the import and synthesis of compatible solutes (Hoffmann and Bremer, 2017).

Synthesis of the S1290 asRNA is strictly dependent on SigB, the master regulator of the general stress regulon of *B. subtilis* (for review, see Hecker et al., 2007). Like other members of the SigB-regulon (Nicolas et al., 2012), S1290 is transiently expressed in response to salt stress, one of the strongest inducers of this group of genes. The S1290 asRNA originates from a promoter present in *opuBD*, the fourth gene of the *opuB* operon, and covers almost the entire coding region of *opuB* (Figure 1B). An asRNA corresponding to S1290 does not exist for the *B. subtilis opuC* operon (Nicolas et al., 2012), despite the fact that the genes for the closely related but functionally different OpuB and OpuC transport systems have evolved through a gene duplication event (Kunst et al., 1997; Kappes et al., 1999). We found that, although the amino acid sequences in the respective segments of the OpuBD and OpuCD integral membrane proteins are identical, only the *opuBD* gene possesses the −35 and −10 sequences of a consensus SigB-dependent promoter (Petersohn et al., 2001; Nicolas et al., 2012; Figure 3A). Its mutational inactivation prevents the synthesis of the S1290 asRNA.

We discovered that the S1290 asRNA is responsible for a delayed induction of *opuB* transcription after a suddenly imposed osmotic upshift. Depending on the degree of the imposed osmotic stress (0.4 and 1 M NaCl, respectively), expression of the S1290 asRNA retards the increase in *opuB* transcript levels for up to 60 min. In the absence of the S1290 asRNA, *opuB* transcription occurs rapidly in response to the osmotic cue. Through asRNA-mediated regulation, the amounts of the components of the OpuB ABC transporter will in all likelihood be negatively affected, and hence the maximal transport capacity of the OpuB system cannot be fully attained in the early phase after sudden imposition of salt stress. Since the time-delayed osmotic induction of *opuB* expression is only observed when the S1290 asRNA is expressed from the antisense strand of the native *opuB* locus, it can be assumed that antisense regulation of *opuB* occurs by transcription interference rather than a base pairing-dependent mechanism. A similar regulatory system is found, for example, in the case of the *ubiG* operon of *Clostridium acetobutylicum*, where asRNAs of different lengths that overlap the 3′-part of the coding region were shown to exert their regulatory effect only when expressed in *cis* (André et al., 2008). Regulation by transcription interference rather than by degradation of double-stranded RNA is also supported by the observation that inactivation of the double-strand specific RNase III (Durand and Condon, 2018) did not affect *opuB* sense and antisense transcript levels observed after osmotic upshifts.

Because the S1290 asRNA targets *opuB* and not *opuC*, one wonders what the delayed induction of *opuB* transcription might accomplish in terms of the ecophysiology of osmotically stressed *B. subtilis* cells. The soil, one of the major habitats of *B. subtilis*, is an ecosystem with rather harsh conditions (Earl et al., 2008; Mandic-Mulec et al., 2015). Hence, any measure that the cell can take to adjust in an energy-efficient manner to the prevailing conditions will aid its survival and growth. Most of the osmostress protectants that *B. subtilis* can use are produced by plants (Hoffmann and Bremer, 2017). These compounds are present in very low concentrations in the soil and root exudates (Warren, 2013, 2014; Bouskill et al., 2016; Webb et al., 2017) and they are also subjected to a high turn-over (Warren, 2019), probably because many microorganisms can also use them as nutrients (Welsh, 2000). Hence, there is in all likelihood a strong competition for the acquisition of these compounds by microorganisms, and consequently, high affinity transporters such as OpuB and OpuC (Hoffmann and Bremer, 2017) are needed to scavenge them effectively under osmotic stress conditions.

Because of different energetic requirements, the import of pre-formed osmoprotectants provides a considerable advantage over their *de novo* synthesis or their production from precursor molecules (Oren, 2011). In line with this premise, the size of the osmoadaptive proline pool of *B. subtilis*, an energetically costly to produce (Akashi and Gojobori, 2002) and the only compatible solute that *B. subtilis* can synthesize *de novo* (Whatmore et al., 1990; Brill et al., 2011), is reduced in response to the environmental availability and import of other compatible solutes (e.g., glycine betaine) (Hoffmann et al., 2013).

When one interprets the delayed induction of *opuB* transcription via the S1290 asRNA in the above-described bioenergetic and ecophysiological framework, it appears that during the early adjustment phase of *B. subtilis* to acute osmotic stress, the cell prefers to initially rely on the transport activity of the promiscuous OpuC system. This would allow an energy efficient and rapid relieve from osmotic stress by the import of most of the pre-formed compatible solutes that *B. subtilis* can use and which it will find in its various habitats (Warren, 2013, 2014; Webb et al., 2017). Plant material is rich in choline as well (Chen et al., 2013) and choline can be imported with similar high affinities via the osmotically inducible OpuB and OpuC ABC transporters. However, choline does not convey immediate relieve from osmotic stress because choline is not a compatible solute *per se*, but needs to be converted to glycine betaine to confer osmostress protection (Boch et al., 1996). Most likely, delayed induction of *opuB* ensures preferential uptake of pre-formed compounds such as glycine betaine. In line with this, the OpuC transporter possesses a higher affinity for glycine betaine as compared to choline (K_*m*_ values of 5 and 27 μM, respectively) (Teichmann et al., 2017). Moreover, in the presence of glycine betaine and choline, S1290-mediated regulation prevents strong induction of the GbsR-dependent *opuB* and *gbsAB* operons. It was shown earlier that choline-mediated release of GbsR from the *opuB* and *gbsAB* operator sites is counteracted by intracellular accumulation of glycine betaine (Nau-Wagner et al., 2012), thereby preventing excessive formation of glycine betaine by osmotically stressed cells. Our data indicate that GbsR-mediated regulation is also sensitive to the presence of glycine betaine in the environment and that the intracellular glycine betaine to choline ratio accomplished by retarding choline import can diminish the release of GbsR and in turn prevent excessive synthesis of the OpuB transporter and choline converting enzymes. Hence, in concert with the GbsR repressor, SigB-dependent regulation of the *opuB* operon ensures optimal use of compatible solutes and energetic resources available to salt-stressed *B. subtilis* cells.

The transcriptional profile of the S1290 asRNA closely corresponds to that of other SigB-dependent genes, including a strong yet transient up-regulation in response to a sudden salt-shock (Boylan et al., 1993; Völker et al., 1995; Young et al., 2013). Indeed, our mutational analysis of the predicted SigB-dependent promoter in *opuBD* proved that the synthesis of the S1290 asRNA is strictly dependent on the alternative transcription factor SigB. Moreover, we showed that, when *B. subtilis* is grown in the presence of choline, salt-dependent induction of S1290 is reduced and *opuB* expression increases in response to a suddenly imposed salt stress, albeit significantly less than in the mutant lacking the S1290 asRNA. The decrease in S1290 induction was shown to relate to intracellular accumulation of glycine betaine known to occur during growth of *B. subtilis* in SMM (Hoffmann et al., 2013). Imported or synthesized glycine betaine can apparently alleviate salt stress perception and SigB activation as shown by genome-wide analysis of the transcriptional profile of SigB-controlled genes. The role played by the S1290 asRNA in controlling the onset of *opuB* expression under acute osmotic stress conditions, revealed an integration of physiologically specific adjustment systems to acute osmotic stress with the general stress response network of *B. subtilis* at a level deeper than previously appreciated.

## Materials and Methods

### Construction of *B. subtilis* Mutant Strains

All *B. subtilis* strains used in this study are listed in Table 2 the oligonucleotides are listed in Table 3. The *B. subtilis* strain BHR006 (Δ*txpA* Δ*yonT* Δ*rnc*) was constructed by sequential deletion of the toxin genes *yonT* and *txpA* (Durand et al., 2012) followed by deletion of *rnc*, using a modified two-step PCR method for synthesis of linear DNA fragments carrying a resistance marker flanked by sequences homologous to the upstream and downstream regions of the respective gene (Wach, 1996). The corresponding fragments were amplified using the primer pairs listed in Table 3 and fused to the respective gene mediating resistance to spectinomycin (spc), phleomycin (phleo) or kanamycin (km). The resulting fragments were used to transform competent *B. subtilis* BSB1 cells (Harwood and Cutting, 1990; Konkol et al., 2013), with subsequent selection for antibiotic-resistant colonies. The correct insertion of the resistance marker and absence of point mutations in the upstream and downstream regions were verified by sequencing (Eurofins Genomics, Ebersberg, Germany). To avoid potential polar effects, all deletions are in-frame replacements of the gene coding sequences by the respective resistance gene sequence, leaving the chromosomal context intact. The *gbsR* deletion mutant strain TMB405 was constructed by transforming *B. subtilis* BSB1 with chromosomal DNA (0.2 μg) of *B. subtilis* JBB8 (*gbsR::neo*, Boch et al., 1996).

**TABLE 2:** *B. subtilis* strains used in this study.

*Strain*	*Genotype*	*Reference*
BSB1	*trp*^+^	Nicolas et al., 2012
BSB1 Δ*sigB*^*a*^	*trp*^+^ Δ*sigB::HindIII-EcoRV::cat*	Igo et al., 1987
BHR006	*trp*^+^ Δ*tpxA::spc* Δ*yonT::phleo* Δ*rnc::km*	This study
BHR010	*trp*^+^ *yvaQ-spc* P_*S*1290_ inactivation	This study
BHR018	*trp*^+^ *yvaQ-spc* P_*S*1290_ inactivation (*amyE*::*opuBA’-BB-BC-BD cat*)	This study
TMB405	*trp*^+^ Δ*gbsR::kan*	This study

*^a^Strain BSB1 ΔsigB was generated by transformation of B. subtilis BSB1 with chromosomal DNA of B. subtilis ML6 (ΔsigB::HindIII-EcoRV::cat).*

**TABLE 3:** Oligonucleotides used in this study.

*Name*	*Sequence (5′−3′)*
**Oligonucleotides for deletion of *txpA***	
txpA_up_for^1^	CTTAAGTCTTTCAGCATTGCC
txpA_up_rev^c,1^	**TATTAATTTGTTCGTATGTATTCA**TAATTTCACCTCCTTTCATATTC
spc_for^a,2^	ATGAATACATACGAACAAATTAATA
spc_rev^a,2^	TTATAATTTTTTTAATCTGTTATTT
txpA_do_for^c,3^	**AAATAACAGATTAAAAAAATT**ATAATTTAAAAGCTAGAGTGCTG
txpA_do_rev^3^	GCTGTTCTAGATGACGCCTC
**Oligonucleotides for deletion of *yonT***	
yonT_up_for^4^	TGATATTGCTCGCAGCTTGC
yonT_up_rev^c,4^	**GCCGGGATAGACTGTAACAT**TATGTACACCTCCTTTCCTATG
phleo_for^b,5^	ATGTTACAGTCTATCCCGGC
phleo_rev^b,5^	CGCGCCCGATTGCTGAACAG
yonT_do_for^c,6^	**CTGTTCAGCAATCGGGCGCG**TGAGAGCTAAGCTAAAGGGG
yonT_do_rev^6^	AGTTTCATCCAGGATAAGAG
**Oligonucleotides for deletion of *rnc***	
rnc_up_for^7^	AGAGACAATCTCATCATGAG
rnc_up_rev^c,7^	**GGTCCATTCACTATTCTCAT**AGTAACCTCCATAGGCACATC
km_for^b,8^	ATGAGAATAGTGAATGGACC
km_rev^b,8^	GATTAACAATTATTAGAGGTC
rnc_do_for^c,9^	GACCTCTAATAATTGTTAATCCAGCACGCTGCTCAGGAAGC
rnc_do_rev^9^	CAAGCTCTTCTTCTTTTGCC
**Oligonucleotides for base substitution in the S1290 promoter region**	
yvaQ_up_for^10^	AGCAGGCCCAAGACGCGGTG
yvaQ_up_rev^c,10^	**GTGTTCATTCATGGACCTCCTT**TAAATCGTAAACTGACCCATC
spc_for^a,11^	AAGGAGGTCCATGAATGAACACGTACGAGCAGATC
spc_rev^a,11^	TTACAACTTCTTTAAGCGGTTGTTC
P_*S*1290__*for*^c,12^	**CAATACTGATAATGCCTGTGTAC**GTATTACGAATAATCGGCAGCAGACTGTACAGAAATAATGATAGAATC
P_*S*1290__fusion_rev^13^	GTACACAGGCATTATCAGTATTG
opuBD_up_for^c,13^	**GAACAACCGCTTAAAGAAGTTGTAA**AGAAAAAAGAGGCTGGACTCCAGCCTCTTTTTCTATTCTATGCAGCTGATTGAACATG
opuBD_do_rev^12^	CTCAAAGGAAACGGCTATCAGG
**Oligonucleotides for integrating promoterless *opuB* operon into *amyE***	
ycgB_up_for^14^	CATCCGGAATGCTCATGCCG
opuBA_up_rev^c,14^	**TGATAAGCTGTCAAACATGCTGACATTAGAAAATGTCT**CG
px.vector_for^c,15^	**AGACATTTTCTAATGTCAGCATGTTTGACAGCTTATCA**TCGGCA
amyE.back_rev^15^	AATGGGGAAGAGAACCGCTTAAG
**Northern blot probes**	
opuBC_for	GATTAAAAATCTTGGCTCCA
opuBC_rev.T7^d^	GAAATTAATACGACTCACTATAGGGAGAGATACGGTCTCTAAATGGTA
opuCC_for	GGCTTGGCGCGTTTGCTCTC
opuCC_rev.T7^d^	TAATACGACTCACTATAGGGAGGCAGCCAAGCATTGTCGACGCC
S1290_for	CATTAAGACGGCCAGCATAG
S1290_rev.T7^d^	GAAATTAATACGACTCACTATAGGGAGACGTCTGTCGTTGCCAAGGAA
gbsA_for	ATGAGTCAAACATTATTCAT
gbsA_rev.T7^d^	GAAATTAATACGACTCACTATAGGGAGAATAATTTTGCTTTCTGAATC

*^a^Plasmid pUS19 carries the spectinomycin resistance gene (LeBlanc et al., 1991; Benson and Haldenwang, 1993). ^b^Plasmid pDG148 carries the phleomycin and kanamycin resistance genes (Stragier et al., 1988). ^c^Overlap sequences to facilitate fusion PCR are marked in bold. Primer pairs are labeled in consecutive numerical order. ^d^T7 sequences are underlined.*

For inactivation of the S1290 SigB-type promoter, the −35 core promoter region was changed in two positions from GTTTCG to ATTACG that still encodes for arginine (CGT) and asparagine (AAT) and the −10 region was changed in four positions from GGGAAT to GACTGT that still encodes for tyrosine (TAC) and serine (AGT) at the respective positions of *opuBD* on the opposite strand (see Figure 5A). To generate the P_*S*1290_ mutant, a linear DNA fragment carrying a spectinomycin resistance gene flanked by sequences homologous to upstream and downstream regions of the S1290 promoter was synthesized using the listed primer pairs (Table 3). The upstream sequence corresponds to *yvaQ*, and the spectinomycin resistance gene with a Shine-Dalgarno sequence was placed behind the *yvaQ* gene, creating a transcriptional fusion terminated at the original *yvaQ* terminator to avoid read-through into the *opuB*-operon. The base substitutions were introduced into the downstream fragment via primer P_*S*1290__for. The four overlapping fragments were merged by fusion PCR and used for transformation of competent *B. subtilis* BSB1 cells. Antibiotic-resistant transformants were selected and confirmed by sequencing (Eurofins Genomics, Ebersberg, Germany). The BHR018 mutant was constructed by integrating the *opuB* region without the *opuB* promoter into the *amyE* gene of strain BHR010. Genomic DNA of a *B. subtilis* strain in which the complete *opuB* region was integrated into *amyE* by use of the cloning vector pX (Kim et al., 1996) served as PCR template. Two DNA fragments containing (i) sequences homologous to the upstream region of the integration site and the *opuB* coding sequence and (ii) the *cat* gene of the pX vector and sequences homologous to the downstream region of the integration site were synthesized using primer pairs listed in Table 3. The two fragments were combined by fusion PCR (Wach, 1996) and used to transform competent *B. subtilis* BHR010 cells. Antibiotic-resistant transformants were selected and confirmed by sequencing (Eurofins Genomics, Ebersberg, Germany).

### Media and Growth Conditions

*Bacillus subtilis* transformants were selected on lysogeny broth (LB) agar plates containing spectinomycin (200 μg ml^–1^), phleomycin (2 μg ml^–1^) or kanamycin (2 μg ml^–1^).

For stress experiments, *B. subtilis* strains were cultivated in Spizizen’s minimal medium (SMM) (Harwood and Cutting, 1990) with 0.5% (wt/vol) glucose as carbon source. Cultures were inoculated from exponentially growing pre-cultures to an optical density at 600 nm (OD_600_) of 0.1 and propagated at 37°C under vigorous shaking. To induce hyperosmotic stress, osmolarity was increased by addition of NaCl (pre-warmed 4 M stock solution prepared in SMM) at OD_600_ of 0.3. When indicated glycine betaine or choline were added to the growth medium from 1 M stock solutions.

Samples for RNA preparation were collected at different time points after an osmotic upshift as indicated. Cells were harvested by addition of ½ volume of frozen killing buffer (20 mM Tris/HCl [pH 7.5], 5 mM MgCl_2_, 20 mM NaN_3_) and subsequent centrifugation for 3 min at 8.000 *g* and 4°C. After discarding the supernatant, cell pellets were frozen in liquid nitrogen and stored at −80°C.

### Northern Blot Analysis

Total RNA was prepared by acid-phenol extraction after mechanical cell disruption as described previously (Nicolas et al., 2012). The quality of the RNA was assessed by means of an Agilent 2100 Bioanalyzer (Agilent Technologies, Santa Clara, CA, United States) according to the manufacturer’s instructions. Northern blot analysis was performed as described before (Homuth et al., 1997). Transcript sizes were determined using the RiboRuler High Range Ladder (Thermo Fisher Scientific, Waltham, MA, United States) and the digoxigenin-labeled RNA Molecular Weight Marker II (Roche Holding AG, Basel, Switzerland). The bands of the unlabeled RNA size standard were marked directly on the membrane, thereby becoming visible as negative bands during detection that were then indicated by lines in the northern blot images. Quantification of the 3.5 kb *opuB* transcript and the 1.2 kb S1290 transcript was performed using Image Studio Lite (version 5.2.5, LI-COR Biosciences, Lincoln, NE, United States).

### Transcriptome Analysis

35 μg of total RNA from two biological replicates per condition were DNase-treated using the RNase-Free DNase Set (Qiagen, Hilden, Germany) and purified using the RNA Clean-Up and Concentration Kit (Norgen, Biotek Corp., Thorold, ON, Canada). After quality control (Agilent 2100 Bioanalyzer), 5 μg of the purified RNA were subjected to microarray analysis. Synthesis and fluorescence labeling of cDNA followed a strand-specific method using the FairPlay III Microarray Labeling Kit (Agilent Technologies, Santa Clara, CA, United States) and actinomycin D (Calbiochem, Merck KGaA, Darmstadt, Germany) (Nicolas et al., 2012). 100 ng of Cy3-labeled cDNA were hybridized to the microarray following Agilent’s hybridization, washing and scanning protocol (One-Color Microarray-based Gene Expression Analysis, version 5.5). Data were extracted and processed using the Feature Extraction software (version 12.1). For each gene, the median of the individual probe intensities was calculated and further data analysis was performed using Genedata Analyst^TM^ software (Genedata AG, Switzerland) and R 3.5.0 (R Core Team, 2018). The microarray data set is available from NCBI’s Gene Expression Omnibus (GEO) database (accession number GSE141882).

## Data Availability Statement

The datasets generated for this study can be found in the NCBI’s GEO database, accession number GSE141882.

## Author Contributions

HR, EB, UV, and UM designed the study and wrote the manuscript. HR, AR, TH, EH, AS, UV, and UM designed the experiments. HR, AR, TH, EH, and AS performed the experiments. HR, AR, TH, EB, UV, and UM interpreted the data. HR, EB, UV, and UM.

## Conflict of Interest

The authors declare that the research was conducted in the absence of any commercial or financial relationships that could be construed as a potential conflict of interest.

## Footnotes

1 http://genome.jouy.inra.fr/cgi-bin/seb/index.py

2 http://genome.jouy.inra.fr/cgi-bin/seb/viewdetail.py?id=S1290_3460206_3462957_1
